# First Report of the Commissioners for Inquiring into the State of Large Towns and Populous Districts

**Published:** 1844-10-01

**Authors:** 


### Medico-Chirurgical Review,

### No. LXXXII.

[No. 42 of a Decennial Series.]

JULY 1, TO OCTOBER 1, 1844.

### First Report of the Commissioners for inquiring into the State of large Towns and populous Districts. Presented to both Houses of Parliament by Command of Her Majesty.

The subject of the Report before us is the wide spread existence of removable causes of disease, and the best mode of securing their removal.

That prevention is better than cure is in no case so true as in that of medicine. Great as is the utility of efficiently treating disease—the preservation of health is greater still. The cure of a disease, as it is called, is often the partial removal only of an existing evil—its prevention is its complete avoidance. It rarely happens that the subject of severe sickness, however complete may be his apparent recovery, escapes without some permanent injury to his constitution, which either lowers the standard of his health, and adds, practically, years to his constitutional age, or, at least, renders him more prone than he would otherwise be to subsequent attacks of the like or analogous disorders.*

Then again, how often does it happen that complete recovery is not the issue of the best directed remedial treatment. Not rarely death claims his victim before his naturally appointed time, and still more frequently the occurrence of a severe attack is but the commencement of a series of tormenting and protracted sufferings. By the prevention of disease, then, not only is immediate risk and pain, and anxiety avoided, but permanent injury prevented, and unquestionably a large addition made both to the duration and the desirableness of existence.

Until a comparatively recent period this most important subject,—attention to the general health of the community—had met with but little attention either from the profession or the public. What is everyone's business, is no one's business, and this has been no exception. Our knowledge, even of the exciting causes of particular diseases, is in a very unsatisfactory state, that of the remote and predisposing causes, of the reasons for the prevalence of particular classes of disease among certain portions of the population, is still more imperfect. The contrast between our ignorance on these subjects and the advanced state of our knowledge in pathology and practical medicine and surgery is very remarkable. Nor is it surprising. Knowledge on the latter subjects is required for our ordinary business; by it is our road to fame and fortune, and by its employment we earn our daily bread. For the other sort of knowledge there is

* This is so well known that many insurance offices will not accept the lives of those who have been previous sufferers from any serious disease, and all make it an important object of enquiry in estimating the risk of each particular life.

little demand, and consequently but a small supply. For without in the least insinuating that the members of our noble profession are habitually actuated by merely mercenary motives, they, like other men, pay but little attention to that, which, however valuable, is not useful to themselves; and a subject of such extent and difficulty as that of the nature and mode of removal, of removable causes of disease, will never be thoroughly understood, and still less will such information be widely diffused, until a number of men are engaged in acquiring and employing the knowledge *as a profession.* That not being the case, we ought rather to be surprised that our knowledge is so extensive as we find it, than that it is so confined as we confess it to be.

The Commissioners, whose Report we are examining, were appointed by Her Majesty's Letters Patent, dated may 9th, 1843, "to inquire into the present state of large towns and populous districts in England and Wales, with reference to the matters hereunder specified :—

"The causes of disease among the inhabitants.

"The best means of promoting and securing the public health under the operation of the laws and regulations now in force, and the usages at present prevailing with regard to—

"The drainage of lands;

"The erection, drainage, and ventilation of buildings;

"And the supply of water in such towns and districts, whether for purposes of health, or for the better protection of property from fire, and—

"How far the public health and the condition of the poorer classes of this realm, and the salubrity and safety of their dwellings, may be promoted by the amendment of such laws, regulations and usages."—*Report, p.* vii.

The Report, with its evidence and appendices, appears in the form of a large blue book, of more than seven hundred pages. It is clear that we cannot present even an outline of so vast a mass of matter, and all that we shall attempt is to abstract enough to establish the following points. First—the wide diffusion and vast importance of the evils to be remedied. Secondly—that the causes of the evils are removeable. Thirdly—that the cost of their removal would impose no inordinate burden upon those who would have to bear the expense of, as well as receive, the benefit to be conferred.

1*st. The wide Diffusion and Extent of the Evil to be remedied.*

Upon this point we may be very brief. The wide diffusion of the evil of excessive unhealthiness of towns is established by the fact, that out of 40 of the largest towns in England, four only have a rate of mortality not exceeding that of the average of the country; which average is itself raised by this excessive, and, as we shall shortly try to prove, artificially high mortality. The mortality of healthy districts is about two per cent. Eight only of the forty towns above alluded to have a rate of mortality less than two and a half per cent. while five have a rate exceeding three per cent.; and in one, Liverpool, the mortality has been three and a half per cent. In other words, while in a healthy district twenty out of a thousand inhabitants die annually, and the average duration of life is more than forty years, in five of these large towns thirty or more out of every thousand perish in the same time, and the average duration of their lives is reduced by one third of its natural period. It requires no argument to prove the incalculable importance of measures tending to diminish this fearful sacrifice of life, to alleviate the injury by which it is accompanied, and to extend to its natural term the period of existence for vast multitudes of our fellow creatures.

We will, however, present another illustration. In the fifth Report of the Registrar-General, at page 400, a table is published which well deserves attentive study. It is too long for extract, we must content ourselves with giving some of the results. It is an abstract of the deaths registered in the year 1841, in the districts of 25 towns, in the Metropolis, and in Cornwall, Devonshire, Dorsetshire, Somersetshire, Wiltshire, Essex, Gloucestershire (except Bristol and Clifton), Herefordshire, Norfolk (except Norwich), Suffolk, Sussex, and Westmorland. The population of the Metropolitan and town districts was, in 1841, rather more than three and a half millions (3,759,186), that of the rural districts was rather less (3,440,501). The disproportion in the number of deaths, however, far exceeded this small disproportion in the amount of population, being 96,999 in the town districts, and only 65,575 in the rural districts, being a difference of 31,424. In other columns the comparison is made of the mortality among exactly equal civic and rural populations. The calculation is made of the number of deaths from all causes occurring in 1841, among a population of 1,000,000 of both town and country districts: of the former 25,803, of the latter 19,060 died in the year 1841. For reasons into which we will not at present stop to enquire, the mortality in towns appears to have been less in 1841 than usual; and in the average of the four years 1838-39-40 and 41, 27,073 died annually in each million in the town districts, while 19,300 only died in each million in the country districts.

We are now in a condition to form an estimate, though still an imperfect one, of the vast extent of the evil for which we must seek a remedy. If it really be, as many believe it is, quite possible by proper sanatory precautions, to reduce the mortality of towns to the same rate as that of the country districts, it follows that we have it in our power to diminish the number of deaths in the four millions of large town population by more than thirty thousand a year. But the benefit of improved sanatory precautions, though most urgently required in large towns, will not be confined to them. All parts of the country will participate, and we may estimate the number of lives which may probably be annually saved, at a number considerably exceeding that which we have named.

It is with difficulty that the mind receives the idea of numbers larger than it is accustomed to deal with. A population of thirty thousand is that of a considerable town, such for instance as Derby, Lancaster, or York. Let us imagine one of these towns to pour forth all its inhabitants; let every dweller from cellar to garret appear; let every street, every alley, give up its living horde; let all, male and female, of every age, from the new-born babe to the octogenarian tottering on the brink of the grave, join in the vast crowd, to give us some slight idea of the enormous multitude which we represent by the words THIRTY THOUSAND LIVING SOULS. And yet this number, vast as it is, is but a part of those who, in all human probability, might be annually saved by proper attention to the circumstances necessary for healthful existence.

But this vast unnecessary loss of life, together with the melancholy amount of suffering by which it is preceded, is a part only of the evil resulting from faulty sanatory arrangements. Each of these unnecessary deaths represents a family unnecessarily bereaved. Early orphanage with all its risks, premature widowhood with all its privations, are not among the least of the evils it is within our power to lessen. Again, it is probable that sickness and mortality have a pretty constant ratio to each other,* and that by the same means by which we diminish fatal disease, we shall in like proportion lessen that which is not destructive of life. If so, we may calculate upon a diminution of physical and mental suffering beyond that already indicated. According to the experience of the

* That this ratio is nearly constant is probable, but by no means certain. Any difference, however, there may be, is likely to be in favour of our argument. It is certain that many cases run their course, whether for recovery or death, with greater rapidity in country constitutions, and such cases will tend to diminish the ratio of sickness to mortality in the country. It is probable, however, that the ratio is sufficiently constant for the substantial correctness of the argument in the text.

Manchester Dispensaries, (the only returns we happen to have at hand just at present,) there are 28 cases admitted for each death: if this be about the general proportion, and if the number of general cases be proportionate to the number of fatal cases, those sanatory measures which shall diminish the deaths among a population of four millions by thirty thousand, will diminish the number of cases of sickness by 28 times as much, or by more than eight hundred thousand.* It is absolutely impossible to form an adequate idea of the amount of suffering unnecessarily endured by the occurrence of such a number of avoidable cases of disease.

Enough has now been said to prove and to illustrate the magnitude of the evil we have to contend against, and we now pass on to the proof of that which we have hitherto been tacitly assuming, namely—

2. *That the Causes of the Insalubrity of Towns are removable*, or at least are susceptible of very considerable alleviation. An essential preliminary to this part of the question is the enquiry, what are the circumstances of a town-life which render it less favourable than a rural life to health and longevity? It may now be considered to be an established truth, that the possession or deprivation of the comforts of life exerts a most powerful influence upon health. Of these the most immediately affecting health are the quantity and quality of food, and probably the next most important is the degree of purity of the air we breathe. It is however doubtful which of these two great elements of healthfulness or of sickness exerts the more powerful influence. Certain it is, however, that either impurity of the air, or deficiency or unwholesomeness of food, will soon show its baneful influence in increased sickliness and a higher rate of mortality among those exposed.

The inhabitants of the towns have, for the most part, a greater command over the comforts and necessaries of life than the mass of the rural population. The general rate of wages for the former is higher, while the expense of food, clothing, lodging, and firing, is much the same as in the country; and the ease of access to efficient medical assistance when illness or accident may occur, is a further advantage which they possess; and yet we find among them a rate of mortality higher, in the ratio of nearly three to two, than among a rural population. Doubtless the circumstances to which this disparity must be principally attributed are the general impurity of the air, arising from the deficiency of ventila-

---

* We would not be understood as presenting this as an accurate calculation of the proportion of sickness to mortality. We have no proof that they have a *constant* ratio to each other. It is evident, however, that whatever diminishes the number of deaths must produce a still greater diminution in the number of cases of sickness; though it may not be true that the latter diminution will be proportionate to the former. If, however, it be so, as is not improbable, we may estimate the diminution by another process. According to Mr. Farr, the average number in England constantly ill is about three per cent. The rate of mortality in England is about 2.2178 per cent., that is about 22,178 in a million die annually, and about 30,000 are constantly sick on the average. But in the country-districts, where 19,300 in the million die annually, 26,110 only will be, on the above supposition, constantly sick; while in town-districts, where 27,073 in the million die annually, 36,600 will be constantly sick; or in populations of four millions each, there will be forty thousand more, on the average, constantly suffering from sickness in the districts of high, than in those of low mortality. Supposing the average duration of the cases of sickness to be about three weeks, or $\frac{3}{52}$ of a year, the number of annual cases of sickness will be, according to this calculation, more than seven hundred thousand: a result not very widely different from that given as an approximation in the text.

tion, and the existence of collections of putrefying organic matter, joined very frequently to a deficiency in the supply of pure water for drinking, for cooking, and for personal and domestic cleanliness; to which sources of unhealthiness must be added, want of healthful out-door exercise, and habits of dissipation and intemperance, which are perhaps more indulged in when large numbers are gathered together, and which seem at least more powerfully to produce ill consequences under such circumstances.

Three of these great causes of mischief to which a town-population is peculiarly exposed, are almost completely under our control; namely, the general impurity of the air we breathe in towns, the deficient ventilation of the houses, and the deficient supply of pure water; while it is quite possible very considerably to increase the facilities for that invigorating exercise and useful relaxation, the want of which is a direct cause of diminished healthfulness, by the privation of that employment of our physical organs, which is necessary for their proper development, and is also a powerful indirect cause, by almost driving those who have no access to innocent amusement, to seek relaxation from business in the excitement of dissipation.*

It is easy to understand how the air of towns attains that degree of impurity which is so perceptible to those accustomed to breathe the free fresh air of the open country. There is constantly gathering around every inhabited house a quantity of organic refuse, some of which sinks into the soil till it becomes completely saturated, another portion evaporates into the air, but the greater part remains slowly decomposing by the process of putrefaction, until but a small part is left to be carted away, from time to time, as manure to the country. When a house is detached and freely exposed to the passing winds, the small amount of organic accumulation around it so slightly contaminates the air, as to do little or no perceptible mischief. But the case is widely different when a large mass of human habitations are closely congregated together. Now the foulness of the air becomes easily apparent. Its depressing influence is felt by all who have not become habituated to its operation, and upon them its baneful power is shewn by the amount of sickness and the rate of mortality.

The Report before us contains a vast amount of evidence in confirmation of this assertion. It is found in those towns where a large proportion of the inhabitants live in the midst of dirty close streets and courts, where, from deficient drainage or inefficient scavanging, unusually large accumulations of organic refuse are allowed, that in such places the rate of mortality is *universally* high. But such districts are almost exclusively inhabited by the poor; and poverty means insufficient food, clothing, firing, lodging, undue exposure to the inclemency of the weather, excessive labour, and depression of mind; and many persons have suspected that the high rate of mortality is due rather to these causes, than to the influence of the impure air which we have already mentioned. That this suspicion is not well founded, will perhaps be inferred from the fact that the rate of mortality, though certainly much influenced by the poverty of the inhabitants, bears a ratio rather to the closeness with which they are crowded

---

* We rejoice to learn that the importance of provision for healthful recreation is becoming more and more generally acknowledged, and better than merely acknowledged, the conviction is being acted upon. Within the last few months we have heard of efforts making in several of our large towns, especially in the manufacturing districts, to provide parks, walks, and play-grounds for their crowded populations; and it is said that His Grace the Duke of Norfolk intends to devote fifty acres of his land as a public park for the inhabitants of Sheffield. In Manchester it is proposed to raise the munificent sum of fifty thousand pounds for this purpose by private subscription, and we are told it will be done. These are probably among the first fruits of the enquiry we are now examining.

together, than to the privations they may reasonably be supposed to endure; and also from the known effect of the influences to which we have attributed it. That such inference is correct, is proved by the direct observation of the effect upon the same population, of measures which have lessened the impurity of the air by the drainage and paving of the streets, and the removal from them of the organic refuse by which their whole surface had been previously covered. The most direct evidence of this kind with which we are acquainted is furnished in the report by Mr. Holland of the sanatory condition of Chorlton upon Medlock, a portion of Manchester. Mr. Holland ascertained the number of deaths registered as occurring in twenty streets of that township before and after they had been put in proper condition by paving and sewering. He states:—

"The streets in question are inhabited by about 3,500 persons, among whom the rate of mortality was about 3·1 per cent., or 1 in 32, before the streets were paved, and 2·53, or 1 in 39, since the improvement. There seems to be no reason for supposing that the rank of the inhabitants has altered, or their number diminished. It would appear, then, that the deaths are diminished more than 20 a year out of 110, by putting the streets into proper condition. Nor shall we be surprised at the beneficial effects of such a change, when we recollect that when a street is properly paved and drained, and regularly cleansed, it becomes comparatively easy to keep the houses clean, which before was almost impossible; thence follow habits of personal cleanliness, which before could not be fostered. There is good reason to hope, therefore, that as those habits become strengthened, the beneficial influence will become still more apparent. The facilities afforded to cleanliness are also favourable to economy; clothes and furniture last longer, provisions will keep better, hence there is less waste; and in various ways the scanty income of the poor is made to go further. The different moral effects of living in the midst of dirt or uncleanliness, are too evident to require mentioning."—*Appendix to Report*, p. 63.

We need not be surprised at the effect upon the rate of mortality of a town produced by physical causes of the nature of those above referred to, when we learn to what an extent they are allowed to exist, and the large proportion of the inhabitants who are exposed to their operation. For example, Liverpool has the highest rate of mortality of any town of England; thirty-five in each thousand of her inhabitants perish annually, fifteen or sixteen per thousand more than die in rural districts, and eight or ten per thousand more than die in moderately healthy towns, such as Birmingham, Chester, Leeds, Manchester, &c. all of which have a higher rate of mortality than is at all inevitable. And yet Liverpool has natural facilities of healthfulness greater than any other town so large with which we are acquainted. The explanation of this anomaly is at once supplied by Dr. Duncan's very excellent Report, in which he states that 55,534 out of 223,054 of the inhabitants of the parish of Liverpool in 1841, or more than one-fourth, lived in courts, of which the following is a general description.

"The courts, in which so many of the inhabitants of Liverpool reside, consist usually of two rows of houses placed opposite to each other, with an intervening space of from 9 to 15 feet, and having from six to eight houses in each row. The court communicates with the street by a passage or archway about three feet wide,—in the older courts, built up overhead; and the further end being also in many instances closed by a high wall or by the back or side of an adjoining building, the court forms in fact a cul-de-sac with a narrow opening. Such an arrangement almost bids defiance to the entrance of air, and renders its free circulation through the court a matter of impossibility. When other circumstances, to be afterwards mentioned, are taken into account, such as the dense population and abominably filthy state of many of the courts, it is easy to understand in what way the construction of these dwellings may contribute to swell the mortality of Liverpool. The houses themselves are three stories high, contain three rooms of about 10 or 11 feet square, and being built back to back with the houses of adjoining courts, there is of course no thorough draught. An enumeration of the court and cellar population of the borough was made two years ago, under the authority of the Town-Council, when it appeared there were, in the parish of Liverpool—

| Courts. | | Houses. | | Inhabitants. |
|---|---|---|---|---|
| 1,982 | containing . . . | 10,692 | and . . | 55,534 |

that is to say, more than one-fourth of the whole parochial population, or more than one-third of the working classes were residents in courts. With regard to the character of these courts, it appears from the Report of the Corporation Surveyors, that 629, or nearly one-third, were closed at both ends; 875, or less than one half, were open at one end; and only 478, or less than one-fourth, open at both ends."—*Appendix*, p. 14.

But in addition to the one-fourth of the inhabitants who pass their existence under circumstances so unfavourable to health as those above described, we find that nearly one-tenth of the whole number, or one-eighth of the remainder, live in underground apartments, or cellars, of which the following is the description.

" The cellars are 10 or 12 feet square; generally flagged,—but frequently having only the bare earth for a floor,—and sometimes less than six feet in height. There is frequently no window, so that light and air can gain access to the cellar only by the door, the top of which is often not higher than the level of the street. In such cellars, ventilation is out of the question. They are of course dark; and from the defective drainage, they are also very generally damp. There is sometimes a back cellar, used as a sleeping apartment, having no direct communication with the external atmosphere, and deriving its scanty supply of light and air solely from the front apartment.

" The enumeration already alluded to shewed that there were, in the twelve wards forming the parish of Liverpool,

| Inhabited Cellars. | | Inhabitants. |
|---|---|---|
| 6,294 | . . . . . | containing 20,168 |

exclusive of the inhabited cellars in courts (of which there were 621, containing probably 2000 inhabitants). From pretty extensive data which I have in my possession, I should be inclined to think these numbers, both of the court and cellar population, to be under the mark; but, as they profess to be from actual enumeration, I am of course bound to take them as I find them.*

" Of the entire number of cellars, 1617 have the back apartment I have mentioned; while, of 5297 whose measurements are given, 1771, or one-third, are from five to six feet deep, 2324 are from four to five, and 1202 from three to four feet below the level of the street: 5273, or more than five-sixths, have no windows to the front; and 2429, or about 44 per cent. are reported as being either damp or wet.

" The streets inhabited chiefly by the working classes, are on an average perhaps about eight yards in width: they seldom exceed ten and are sometimes not more than five yards across. Each house is usually occupied by two or more families, exclusive of the cellar, and most of the densely peopled lodging-houses are situated in the streets. As a general rule, the houses have no thorough draft, from being frequently built up against the houses in the courts behind."†

---

* Possibly casual lodgers have been omitted in the enumeration.—*Appendix*, p. 14.

† That we are correct in attributing a large part of the excessive mortality of Liverpool to the fact of so large a proportion as one-fourth of her inhabitants living in courts in which free circulation of air is impossible, is justified by

The above extracts give an ample explanation of the high rate of mortality in Liverpool. They prove also beyond all question, that it is quite within our power to diminish very materially that excessive mortality. That it is alike our interest and our duty to exert that power requires no proof.

We have now shewn by two examples (which we consider sufficient, otherwise we might bring forward an almost indefinite number of similar cases), that excessive mortality is produced by the impurity of air arising from organic accumulations, and that such excessive mortality is diminished by the partial removal only of the cause to which it has been attributed. We cannot then resist the conclusion, that if such accumulations and the consequent impurity of air which they produce be entirely removed and completely prevented for the future, a still further diminution may confidently be anticipated; and we cannot doubt that if, by a complete system of drainage and an ample flow of water, all organic refuse were at once swept into the sewers and carried to such distance as that its emanations could be no longer hurtful, a most marked improvement in the public health would inevitably follow. The utility of sewerage has never yet been fully developed. It has been considered hitherto as the means merely of getting rid of the service water only, while its far greater utility in preserving for us the purity of the air has been almost entirely neglected.

It is not common indeed for provision to be made for even the internal drainage of houses at all. According to the Commissioners' Report:—

"The witnesses state that, for the most part, the usages at present prevailing, and the bye-laws in force under the authority of these statutes, have been (until two or three of the Commissions in the metropolis adapted their sewerage to the house drainage), framed with a view to the maintenance of the drainage of surface water only, and without reference to that system which is now admitted by all the medical witnesses to be of the greatest importance to the public health, to the condition of the poorer classes, and the salubrity of their dwellings, namely, house-drainage and sewerage, and the constant removal of all decomposing vegetable or animal refuse, much of which might be effected by means of the proper application of water.

"In some of the larger and most crowded towns, all entrance into the sewers by house-drains, or drains from water-closets or cess-pools, is prohibited under a penalty. In other places, including a part of the metropolis, the entrance of house-drains is commonly deemed the concession of a privilege, subjected to regulations and separate proceedings, with attendant expenses, tending to restrict the use of the sewers for these most important purposes, or to confine the advantage to the wealthy.

"In the local Acts we have examined, and in the bye-laws and usages in force under their authority, the use of the main drains is restricted under penalties from that which, if they were properly constructed and sufficiently supplied with water, it is stated, might be one of their most important services—namely, the

---

general experience, even were direct proof of extra mortality in such localities absent. But we are not entirely without such direct proof, though it is scanty. Mr. Holland has ascertained that out of about 2,500 persons inhabiting open streets, 424 died in five years, or 3·4 per cent. per annum; while out of 1,800 other persons inhabiting courts or streets built up at one end, 387 died in the same time, or 4·3 per cent. per annum. In other words, nine per thousand more died each year among those upon whom this cause of ill health operated most powerfully. In all other respects, except closeness, the streets and houses were as nearly as possible in similar condition, and were inhabited by persons in the same rank of life, namely, the poorest classes. It is possible, however, that the very poorest were driven into the least eligible situations, to which circumstance perhaps some of the extra mortality may be attributed.

rapid, efficient, and economically cleansing of a town of surface refuse, mud, and filth.

"It appears from the unanimous statement of the visiting Commissioners, in addition to an examination of the replies of the fifty towns on the subjects of drainage and cleansing, that in scarcely one place can the drainage or sewerage be pronounced to be complete and good, while in seven it is indifferent, and in 42 decidedly bad as regards the districts inhabited by the poorer classes. The investigations within the several towns of the arrangements for house as connected with street cleansing, present nearly the same results.

"It appears that the local statements and opinions on what is deemed to be good, can only be received with reference to the imperfect standards known in those places. In the answers it is often stated that the drainage of a town is good, where it has been found that only the principal streets have main drains or sewers, and where the houses in those streets are but imperfectly provided with house or branch drains; while the most crowded portions of the town, those most densely inhabited by the poorer classes, are utterly neglected, and have no drainage, the refuse being allowed to accumulate and decompose in open channels and pools, or to run into open and stagnant ditches in the immediate vicinity of the houses.

"The medical witnesses have brought before us facts in support of their strongly urged and unanimous opinion, that no population can be healthy, which live amid cess-pools, or upon a soil permeated by decomposing animal or vegetable refuse, giving off impurities to the air in their houses and in the streets. They state the necessity of preventing all accumulations of stagnant refuse in or near houses, and of substituting a system of house-drainage and cleansing, aided by the introduction of better supplies of water into the houses. They have brought forward instances where the main drains or sewers were tolerably well formed, and subordinate or house-drains attached, but where, from the want of properly directed supplies of water, both house-drains and sewers only acted as extended cess-pools."—*Report, p.* 10.

The evident truth of this is allowed at once. We at once perceive how barbarous is the custom of allowing fæculent matter and foul water, and refuse and filth of all sorts, to lie decomposing on the surface of the ground, poisoning the air with their fœtid effluvia. We unhesitatingly condemn as hurtful, practices such as that described in the Rev. Mr. Clay's Report of Preston, in which an example is given of two rows of cottages between which there is an open cesspool, receiving all the filth from twenty-two houses and privies. In this cesspool the filth remains putrefying, until the receptacle is full, or until the manure-gatherers (the muck-misers as they are quaintly called) find it their interest to carry it away. We see at once that such a practice must be injurious to the health as it is to the comfort of the surrounding inhabitants; and yet it is but a slight exaggeration of the custom which is all but universal—that of keeping in cesspools or middens, closed or open as the case may be, large accumulations of every sort of abomination. Nor is the empoisoning of the surrounding air the only evil, great as that is. The liquid filth often finds its way by filtration to the foundations of the houses, and cellars, and so may pervade the whole dwelling. Nay sometimes it even reaches the springs of water, and renders them foul and even fœtid. When the evil reaches this extent, it is less dangerous than when in a less evident degree, for now another supply of water must be sought. There can be little doubt that the use of water for drinking or cooking so contaminated as to be perceptible to a delicate taste only, but bearable to those accustomed to it, will gradually undermine and destroy the health. Another evil even more injurious is indirectly produced;—that of discouraging water drinking, and fostering the habitual use of stimulating beverages. Dr. T. Clarke of Aberdeen University remarks on this point—

"There is one very obvious consideration as regards the health of the inhabitants, that if you have water not fit for drinking, in which there is matter offensive in any degree, by so much as the water is offensive you lessen the habit of drinking water. Now, you cannot restrict the supply of water to such quality as is naturally repulsive; you cannot thus render the inhabitants abstinent from water, without interfering with the healthful functions of their bodies. It was with no small concern that I learned how few of the inhabitants of London, and especially of the lower orders, drink water. In making my experiments upon these waters, when I enquired of the servants about me how they liked particular waters, it was with particular surprise I discovered that they—generally mere lads—knew nothing about the taste of the water. They are the same sort of persons as would be accustomed to drink water in other places, but they have other beverages here. I should perhaps not speak as to the general habits of the inhabitants, but only of what little I have observed in such circumstances."—*Dr. T. Clark's Evidence, p. 7.*

Some of the direct evils of the present common arrangements are thus pointed out in Mr. Holland's Report.

"Sometimes the midden of a necessary is separated from a house by a wall only, which is not always impervious to liquid. I have known instances, where the wall of a dwelling-house has been constantly wet with fœtid fluid, which has filtered through from a midden, and poisoned the air with its intolerable stench; and the family was never free from sickness during the six months they endured the nuisance. Instances in which fœtid air finds its way into the next dwelling-house are not unfrequent. I know an instance (and I believe there are many such) where it is impossible to keep food without its being tainted for even a single night in the cupboards on the side of the house next to the public necessary, and where the fœtor is offensively perceptible always, and is oppressive in the morning before the door is opened."

In another part of this Report a back street is described "about 4 feet wide and not 300 long in which there are 38 necessaries, each with an open midden. The street being too narrow to admit a cart, the manure has to be carried in wheelbarrows, and much of it is spilt during this tedious operation, and the passage is not generally carefully cleaned afterwards. Each of these middens will contain more than a ton of manure, and as they are not emptied till they are full, there will be on the average in each more than half a ton, or nearly 20 tons of decomposing filth in the very confined space between two rows of houses only about five yards apart, a space, including the yards, of less than 500 square yards. It is evident that the only complete remedy is to prevent the accumulation of manure in such large quantity in the immediate neighbourhood of dwellings, by washing it away into the sewers; that is, by the erection of water-closets in all cases where houses are built closely together."

The adoption of this very effectual remedy need not, we are informed, cost more than two pounds for each house.

We shall not enter upon the consideration of the best mode of completely getting rid of all the refuse matter of towns. The evidence in favour of employing it by means of foul water irrigation appears to us exceedingly strong. This is a point, however, for the determination of the engineer rather than of the physician, and to him we leave it, merely reiterating our conviction, that all accumulations of refuse matter in the neighbourhood of dwellings closely congregated together must be prevented at whatever sacrifice of money, unless we would submit to the far more extravagant sacrifices of comfort, health and life.

A very large portion of the excessive mortality of towns arises from the cause we have already mentioned, an impure state of the air generally: but it is perhaps still more owing to causes existing within individual dwellings. Of the general absence of house drainage we have already spoken; the next great evils are overcrowding, and the want of ventilation, especially in bed-rooms. The difference in the rate of mortality among the inhabitants of well constructed dwellings, and among those living in houses of an opposite description, built back to back, with only one door, and generally two rooms only, is very remarkable. The most unexceptionable evidence on this point which has come under our notice is furnished in Mr. Holland's Report. He has made an analysis of the deaths registered during five years as occurring in each individual street of Chorlton upon Medlock, which is a section of Manchester. The streets were divided according to their condition into three classes, first, second, and third, each of which classes were subdivided into three other classes, according to the character of the dwellings, thus making nine subdivisions. The number of inhabitants of each street was computed from the number of inhabited houses, taking the same ratio in each, between houses and inhabitants as was ascertained by the Census. From the number of deaths occurring in each, the rate of mortality among the inhabitants is easily calculated. The results are exhibited in the following Tables.

"The following table (1) exhibits the proportion per cent. of each class of streets and of houses, in which the rate of mortality has been, during the last five years, below 2 per cent. per annum, between 2 and 3 per cent., and above 3 per cent.

TABLE I.

| Class of Streets. | Class of Houses. | Proportion per cent. of Streets in which the Mortality has been: Below 2 per cent. | Between 2 & 3 per cent. | Above 3 per cent. | Examples of Classes of Streets and Houses. |
|---|---|---|---|---|---|
| 1st. | 1st. | 50 | 50 | ,, | Oxford-street, Grosvenor-street, Sidney-street, Rutland-street. |
| | 2nd. | 20 | 65 | 15 | Greek-st. Clarendon-st. Chatham-st. |
| | 3rd. | ,, | 33 | 67 | Charles-street, York-st. Chester-st. |
| | 1st. | 54 | 39 | 7 | Angle-street, Carver-st. Prospect-st. |
| | 2nd. | 30 | 30 | 40 | Ann-street, Benton-st. Caygill-st. |
| | 3rd. | 14 | 43 | 43 | Cross-street, Evans-st. Rathbone-st. |
| | 1st. | ,, | ,, | ,, | None of the third-class of streets have first-class houses. |
| | 2nd. | ,, | 50 | 50 | Bell-street, Mark-lane, Charlotte-st. |
| | 3rd. | ,, | 20 | 80 | Medlock-street, Makin-street, Bury-street, Burns-street. |

"Inspection of the above table will show how constantly a low rate of mortality has accompanied a good condition of streets and dwellings, and a high rate of mortality the contrary condition. Among the inhabitants of the first class of houses and streets, it will be seen that in none has the rate of mortality exceeded 3 per cent.; and of the second-class houses and first-class streets, in 1 in 15 only has it risen above that amount. But of the third-class houses, in 67 per cent. of the first, 43 per cent. of the second, and 80 per cent. of the third-class streets, has the rate of mortality exceeded 3 per cent.; while in some particular streets it has been twice that amount. Among the worst are Bury Street, Burns Street, Heyes Street, Dorning Street, Medlock Street, Tebbutt's Court, Wilson Street, Woburn Place, Back Temple Street, Back Kay Street, Allen Street, and Bond Street. All of these streets have some evident defect; the seven first mentioned are not thoroughfares, and are built up, or nearly so, at one end; all of them are unpaved and undrained; the houses are built back to back, and have no back doors. They are all of them little better than courts.

"It will further be observed, that in none of the worst-conditioned streets has the rate of mortality been lower than 2 per cent., whilst those of the best condition a considerable proportion have had a lower rate of mortality; and further, of the worst-conditioned streets, in half of those with second-class houses, the mortality has been less than 3 per cent., while of those with third-class houses four-fifths have had a mortality exceeding that amount.

"These facts present a striking illustration of the ill effects of badly conditioned dwellings and streets, but still more strikingly exhibit the destructive influences of poverty, as shewn by the high rate of mortality among the inhabitants of low-rented houses.

"The following table (2) exhibits the rate of annual mortality in the different classes of streets, of the town part of Chorlton upon Medlock, in the average of the five years ending June 1843. The amount of the population of this part of the township is computed at 24,682; among whom there have been 3235 deaths, or 647 a year, being an average annual mortality of 2.6 per cent., or 1 in 38. The rate of mortality in the different classes of streets will be seen to differ very widely.

TABLE II.

| Class of Streets. | Class of Houses. | Computed Population. | Rate of Mortality. (For examples see Table 1.) |
|---|---|---|---|
| | | | Per Cent. |
| 1st. | 1 | 5153 | 1.95. or 1 in 51. |
| | 2 | 4350 | 2.2. .. 1 45. |
| | 3 | 980 | 2.7. .. 1 36. |
| 2nd. | 1 | 1431 | 1.8. .. 1 55. |
| | 2 | 5094 | 2.6. .. 1 38. |
| | 3 | 2780 | 2.8. .. 1 35. |
| 3rd. | 1 | ,, | ,, ,, ,, |
| | 2 | 820 | 2.8. .. 1 35. |
| | 3 | 4074 | 4. .. 1 25. |

TABLE III.

Exhibiting the Rate of Mortality in different Classes of Streets, the houses being of all rates.

| | | Excess per cent. above first-class streets. |
|---|---|---|
| Streets of First Class | 2.2 per cent., or 1 in 46 | ,, |
| Second | 2.6 ,, 1 39 | 18 |
| Third | 3.7 ,, 1 27 | 68 |

TABLE IV.

Exhibiting the Rate of Mortality among the Inhabitants of different Classes of Houses, the Streets being of all Classes.

| | Rate of Mortality. | Excess per cent. above first-class houses about |
|---|---|---|
| Houses of First Class | 1.9 per cent., or 1 in 52. | ,, |
| Second | 2.5 ,, 1 in 40. | 31 |
| Third | 3.4 ,, 1 in 29. | 78 |

"From comparison of these tables it would appear that the rate of mortality is more influenced by the class of house inhabited, than by the condition of the street. For we find that the mortality in the first, second, and third classes of streets, the houses being of all classes, has been in the proportion of 100, 118, and 168: but in the houses of the first, second, and third classes, the streets being of all classes, that the proportion of mortality has been 100, 131, and 178; there is therefore a greater difference in the rate of mortality among inhabitants of different classes of houses, than in those of streets of different condition. When, however, the evil influences of both badly-constructed dwellings and of badly-conditioned streets operate together, the effect is very striking. For instance, the third-class houses of the first, second, and third-class streets are nearly alike in construction, are about the same size, are charged about the same rent, and are inhabited by about the same class of persons, but the rate of mortality in the third-class houses in first and second-class streets, has been 2.7 and 2.8 per cent. respectively, while in those of the same class of houses, but in third-class streets, the rate has been 4 per cent., or a higher rate of mortality, in the proportion of 10 to 7. I am aware of no circumstances but those connected with the bad condition of the streets, which will account for this great difference.

"It may be thought that these streets are inhabited by a poorer class than the others, but I do not believe that that is the fact, except so far as their poverty is increased by the expenses of sickness and death in their families, and by consequent loss of work. Their incomes while at work must be much the same, for they have the same sort of employments. There seems every reason to hope that if these worst streets were put into good condition, the rate of mortality would fall 25 per cent., or more. The diminution in the rate of mortality has been nearly 20 per cent. in the streets which have been improved; as before stated, it has fallen from 3.1 per cent, or 1 in 32, to 2.53 per cent., or 1 in 39; the effect of a permanent good condition, as we have just seen, appears to be still more beneficial; and there can be no doubt that if the houses, as well as the streets, were put into proper condition, the rate of mortality would fall still more. It is, indeed, unreasonable to expect that the general state of health and longevity of the poor can be raised as high as that of those in more comfortable circumstances by any, the best, sanatory arrangements, but I think the evidence here adduced distinctly shows that the rate of mortality, among the poor certainly, among all classes probably, is unnaturally high, from the operation of removable causes of disease.

"When we find the rate of mortality four times as high in some streets as in others, and twice as high in whole classes of streets as in other classes, and further find that it is all but invariably high in those streets which are in bad condition, and almost as invariably low in those whose condition is good, we cannot resist the conclusion that multitudes of our fellow creatures, hundreds of our immediate neighbours, are annually destroyed for want of the most evident precautions."

We have spoken of the two principal evils attending household arrangements, the destructive effects of which we have here in evidence, as being overcrowding and bad ventilation. The first of these is to a great extent an unavoidable evil. It results from poverty, and is one of the means by which poverty acts destructively upon health. The want of ventilation in houses is to a great extent remediable: and when its vital importance is properly understood, we doubt not the evil will be considerably alleviated.

The next great evil is dirtiness. This arises in a great measure from a deficient supply of pure and soft water. When cleanliness is of very difficult attainment, many persons give it up as hopeless, and a gradual deterioration in the habits of the people is an observed consequence of such difficulty. We have no means of estimating the exact amount of unhealthfulness arising from this cause: we have however some very satisfactory evidence of the beneficial effect of household cleanliness. The police authorities of Edinburgh, alarmed at the rapid spread of fever in the Autumn of last year, empowered Mr. Ramsay their Inspector, to cleanse, fumigate and whitewash at their expense, all dwellings where fever had occurred or was likely to occur, if the occupants made no objection. It appears that no opposition was experienced; that during two months, 303 staircases, 898 rooms, 248 closets and 894 passages; in all 2,443 places were lime-washed and cleansed, at a total expense of about £42. or fourpence farthing each. Up to the date of the Report, one fresh case of fever only had occurred in any of the houses which had been cleansed! We must not expect to prevent the occurrence of fever by such means as this alone, but that we might thus very much diminish both its frequency and its virulence, experience has established beyond all question.

The evidence we have hitherto presented has been to the effect that disease generally is greater in those districts where the causes to which we have attributed its excess, exist in the greatest power, from which we infer that its excess is legitimately attributed to those causes. In confirmation of this inference, or rather in illustration, for confirmation cannot be required, we will now proceed to show that those particular diseases are most prevalent in towns which are well known to be frequently excited and always aggravated, by the same circumstances to which we have attributed the excessive mortality of densely-populated districts—foul air and deficient ventilation. By foul air we would be understood to mean, air polluted either by the products of putrefying refuse, vegetable as well as animal, or charged with the emanations of living bodies, whether in the state of health or of disease; for they are injurious in either condition, though of course the effect of the latter is by far the most powerful. What is the exact nature of these emanations is entirely conjectural, nor is it necessary for our present purpose to enquire: it is sufficient to know that they exist, that they may be, if not dissolved, suspended in the air, and that they act injuriously in proportion to their degree of concentration: indeed they do not appear, when sufficiently diffused, to produce any evil consequences whatever.

As we formerly stated, the rate of mortality among the inhabitants of town districts has been, during four years, 27,072 per million, while it has been only 19,300 per million in those of the country. The following are the causes of death which, according to the return before mentioned, have been most remarkably more destructive in the town districts. These are first the whole class of Zymotic*, (or epidemic, endemic, and contagious) diseases, from which nearly twice as many have perished in the towns as in an equal population in the country.

The following Table shows the numbers per million who have died from the following disorders of this class.

| | Town. | Country. |
|---|---|---|
| Small-pox - - - - - - | 1045 | 507 |
| Measles - - - - - - | 914 | 364 |
| Scarlatina - - - - - - | 988 | 478 |
| Hooping-cough - - - - | 829 | 415 |
| Diarrhœa, Dysentery, and Cholera- | 385 | 196 |

* From Ζυμοω, I ferment. Morbid processes are somewhat analogous to fermentation. Sugar or gluten is excited to fermentation by the addition of a minute portion of matter (yeast) in which that process has already commenced: so also peculiar forms of disease are excited in constitutions prone to such process by the addition of morbific matter arising from existing disease in some cases, or of organic matter in a state of *eremacausis*, in others. See Liebig's Chemistry of Agriculture and Physiology. Chapter xiii.

| | | |
|---|---|---|
| Typhus - - - - - - | 1254 | 998 |
| Erysipelas - - - - - - | 133 | 53 |

There are, perhaps, no diseases which are more influenced by the condition of the air than those of the above list, in which the difference in the mortality in town and country has been so glaringly apparent. As is well known, not only is the number of cases of such diseases diminished by free ventilation, but still more remarkable is the effect of such influence on their severity when they do occur. With respect to Measles, Scarlatina, and Hooping-cough, it frequently happens that, when they occur under good sanatory conditions, they are so slight that they may rather be considered as changes through which the constitution is passing, than diseases: not so, however, is it when they occur under opposite conditions, in unhealthy localities: then indeed, as every practitioner knows by sad experience, they are often most unmanageable and formidable.

The diseases of the nervous system have been, as would be expected, much more destructive in town than in country districts; 4,267 fatal cases per million living, having occurred in the former, and 2,256 in the latter. The following table exhibits the proportion of deaths from particular diseases of this class.

| | Town. | Country. |
|---|---|---|
| Cephalitis - - - - - - | 267 | 111 |
| Hydrocephalus - - - - - | 876 | 334 |
| Convulsions - - - - | 2000 | 852 |
| Delirium tremens - - - | 27 | 10 |

In other diseases of this class the difference is less remarkable. It will be at once perceived that causes likely to produce the above diseases, act with peculiar force upon a town population.

The deaths from diseases of the respiratory organs are excessive in the towns in nearly the same proportion as the general mortality. The deaths have been 7,967 to 5,327, almost exactly 3 to 2.

Asthma and diseases of the heart have been more frequent in the towns in the proportion of 1080 to 406.

The following table exhibits the other diseases in which the greatest disparity has been shewn.

*Annual Mortality to* 100,000 *living.*

| | Town. | Country. |
|---|---|---|
| Teething - - - - - - | 616 | 120 |
| Gastritis and Enteritis - - | 660 | 360 |
| Childbirth - - - - - | 221 | 137 |
| Disease of the Uterus - - | 43 | 23 |
| Old Age - - - - - | 1943 | 2676 |
| Intemperance - - - - - | 19 | 7 |

The cases of deaths from phthisis are 4,463 in the towns, and 3,660 in the country, to each million living, or less than the proportion of deaths from all causes. We do not believe in the correctness of this result; we are sure that the disease is more common in towns than this would indicate. Two circumstances contribute to diminish the apparent *proportionate* mortality from consumption in towns; one is the frequent removal of consumptive patients into the country, and therefore an increase of deaths from phthisis there, the other is the more careful distinction by the better educated town registrars of cases of bronchitis and pneumonia from those of phthisis. We find from the tables that the deaths from these diseases have been 2,304 to 1,088, and we have little doubt that many of these cases would, had they occurred in the country, have been registered as consumption. We think it may fairly be assumed that the registrars in the towns do generally discriminate more carefully, for we find that they have left the cause of death unspecified in one case in 80 only, while the country registrars have done so in one case in 33 : besides it is reasonable to expect that a higher class of men may be procured for the more populous and more remunerative districts.

Upon reviewing the above evidence of the diseases which have been peculiarly fatal in town districts, it will be perceived that they are exactly those which we should, *a priori*, expect to occur, in localities in which the causes to which we have attributed the excessive mortality of towns exist in a powerful degree. First, we find a large excess of zymotic diseases, the frequency of occurrence and severity of which would be certainly increased by the crowding together of many persons in limited space with deficient change of air, and by foul emanations: next, we should expect a large excess of fatal diseases of childhood—these will be principally registered under the heads of teething, convulsions, hydrocephalus, pneumonia, diarrhœa, and the eruptive diseases: and from all of these we find that there has been an excessive fatality in towns. Phthisis is the next disease which we should expect to find peculiarly destructive in badly conditioned districts: that it has been so we do not doubt; why it does not *appear* from the tables to have been peculiarly so, we have already remarked upon.

It will now be perceived that we have, by two distinct routes, arrived at completely correspondent conclusions. We first of all shewed, that wide-spread causes of disease do exist in the districts known to be most unhealthy, and that the rate of mortality was higher where they exist in greatest intensity; and we have now shewn, that the particular diseases which have been most destructively fatal in such districts, are the very same which we should have expected such causes especially to produce. It will not be denied, that to attribute them to these causes is a philosophical inference, nor that to prophesy their diminution proportionate to any diminution of these causes which can be effected, is a certain prognostication. That the causes are to a great extent removable, has become evident as each was mentioned. It now only remains for us to shew:

3. *That the cost of their removal would impose no inordinate burden upon those who would have to bear the expense as well as receive the benefit to be conferred.*

The length to which this article has already extended compels us to make our remarks upon this head very brief. Moreover, though it is an essential part of the question, it is not the part likely to be particularly interesting to professional readers.

If the alterations required were such as to add very considerably to the expensiveness of a poor man's dwelling, the effect would be that the rent being too high for one family to afford, two or more families would crowd into the space which one now occupies, and so the evils that would be produced, would be greater than those which we seek to remedy. This, however, is far from being the case. The expense of the improvements which are most urgently required and which would most materially increase salubrity if adopted, would, when divided amongst the large number to be benefitted, be perfectly insignificant; and we think it would not be difficult to show that the mere pecuniary saving resulting from the improved health, increased energy and prolonged life which may be confidently prognosticated, would far exceed any probable cost. We speak here of the direct pecuniary saving only. The loss which the community now unnecessarily submits to from the existence of removeable causes of disease shows itself in increased suffering, diminished energy, debased morality and premature decay and death of the immediate victims, and in early orphanage, widowhood, bereavement, and not rarely pauperism, endured by their family and friends. It is absurd to think of estimating the value of precautions to diminish evils such as these, in pounds, shillings, and pence; nevertheless it may not be without its value to reckon the money, cost and gain, as a part, though only a subordinate part, of this most important question.

The Report contains several proximate estimates of the expenses likely to be incurred to place towns in a satisfactory sanatory condition. Two of these, one by the Rev. Mr. Clay, of Preston, the other by T. R. Coulthart, Esq. of Ashton, appear to be drawn up with great care; and from these we will compile one of the probable cost of sanatory improvements for a town of a hundred thousand inhabitants.

1. Judging from general experience, it may be expected that about one half of the houses will require to be supplied with water, and that the expense of laying down the pipes will be about 10s. for each house, or £5000. for the ten thousand houses requiring such provision.

2. A larger supply of water is really necessary for health than is generally considered sufficient for household purposes, and a larger reservoir or greater expense for pumping may be required to provide this extra supply. From the evidence it appears that £400. a year is ample allowance for this extra cost.

3. Main sewers.—Assuming that in other towns a proportionate expense must be incurred in drainage as will be required in those from which this estimate is drawn up, we must allow about £5,000. for main sewers, which would be repaid, principal and interest, in thirty years, by an annual charge of £325.

4. The secondary drains, on the same supposition, £39,198. must be allowed to be also repaid in thirty years at £2,560. a year.

5. In most towns the want of house drains is nearly universal. The expense would be about 15s. for each house, or £15,000. for the whole town, which would be repaid at £975. a year.

5. The purity of the air of towns can never be properly secured until the use of water-closets is universal, or nearly so, and all deposits of excrementitious matter near the houses entirely prevented. It is in evidence that cheap but efficacious apparatus of this nature can be fitted up at an expense of £2. which, for a town of twenty thousand houses, would cost £40,000. This sum would be repaid with interest in twenty years, at £3,200. a year, or 3s. 2½d. for each house.

7. Mr. Toynbee, Surgeon of St. George's and St. James's Dispensary, has described a simple apparatus for ventilation, being merely an opening in each room with a valve, which may be constructed at an expense of 3s. or 15s. for a house of five rooms. For a whole town the expense would be £15,000. and would be repaid in twenty years, by £1,200. a year, or 1s. 2½d. each house.

8. The cost of street sweeping, by Whitworth's machine, in Chorlton upon Medlock, which has 28,000 inhabitants, is £650. a year; in the same proportion it would cost £2,321. for a town of 100,000 inhabitants.

9. A considerable outlay might be made, with real economy, for opening out streets and removing obstructions to the free circulation of air. Let us suppose that even so large a sum as £40,000. be so expended, which is far more than ought to be required, or than would be if the welfare of all were justly considered in the general planning of a town, the interest of this sum, at 5 per cent., would be £2,000. a year, or 2s. for each house.

10. No town can be considered to have complete arrangements for securing the health of its inhabitants, where ample provision is not made of places for healthful exercises and recreation. Let us assume that £1000. a year will be required as ground rent and cost of maintenance of public parks, walks, or play grounds.

11. The superintendence of an officer to keep a watch over, and direct the removal of causes of disease, and to point out all available means for securing the general welfare, we consider indispensable for fully carrying out the sanatory measures required. So great, indeed, is the general ignorance and consequent apathy upon subjects such as this, that the hope of effecting any great improvement without making it the professional duty of a competent officer to attend to it, is a mere chimera. The office might be made one of great honour, and need not be one of great emolument. A salary of £300. a year would probably be enough to secure the services of an efficient man.

We have now enumerated all the principal items of expense for the sanatory improvements required, of which the following is the summary.

| | Total expenditure of Capital. | Total annual expenditure, with interest and re-payment of capital. | Number of houses for which the respective improvements are required. | Additional annual rent per house. |
|---|---|---|---|---|
| | £. | £. | | s. d. |
| 1. For water pipes . . . . | 5000 | 400 | 10000 | 9½ |
| 2. For extra supply of water . | | 400 | 20000 | 4¾ |
| 3. Main sewers . . . . . . | 5000 | 325 | .. | 4 |
| 4. Secondary drains . . . . | 39198 | 2560 | .. | 2 6¾ |
| 5. House drains . . . . . . | 15000 | 975 | .. | 11¾ |
| 6. Water-closets . . . . . . | 40000 | 3200 | .. | 3 2½ |
| 7. Ventilating apparatus . . | 15000 | 1200 | .. | 1 2½ |
| 8. Scavenging . . . . . . | | 2321 | .. | 2 3¾ |
| 9. Opening out streets . . . | 40000 | 2000 | .. | 2 0 |
| 10. Ground rent and cost of maintenance of public parks, play-grounds, &c. . . . | | 1000 | .. | 1 0 |
| 11. Inspector of public health . | | 300 | .. | 3½ |

We see then that the above very essential improvements may be paid for at a probable annual expenditure of £14,681; which divided among 100,000 persons is less than three shillings each. This expense for sanatory improvements would be equivalent to an addition to the rental of each house of about 15s. a year, or 3½d. a week, a sum so small as not to be felt burdensome even by the poorest. Nay, even should twice or three times this amount be expended, fully to carry out all the improvements required, the cost would still be far below the mere pecuniary benefit which would inevitably result.

We will now enumerate a few of the principal items of pecuniary saving which may be anticipated. As has been already stated, the ordinary number of deaths in a town population of 100,000, will exceed those of a like number residing in the country by nearly eight hundred a year, (2700—1930=770.) From the evidence we have already adduced, we think it will be allowed that it is not unreasonable to expect that two-thirds of this excess, at least, would be prevented by such sanatory improvements as we have above enumerated. Such a diminution of the deaths would be accompanied by a direct saving of the expenses of more than 500 funerals, which, at the very low estimate of £5. each, amounts to the sum of £2,500. If, as is not improbable, the sickness would be diminished in the same proportion as the deaths, there would be about 14,000 fewer cases of sickness, accompanied by a saving of about as many pounds in money. Supposing each case of sickness to last on the average three weeks, which is about the truth, the loss to the family in expenses of medical attendance, medicine, &c., and in loss of wages of the sick, and of time of the attendants, cannot be less than the twenty shillings we have allowed.

To these large items of expenses save[illegible] must be added perhaps half of what is now spent by the public for the maintenan[illegible] of widows and orphans—expenses which would not be incurred were their natural protectors not cut off by premature death. The amount of this most satisfactory saving would be about £6000. a year.

To these must be added a sum for increased productive power of a more healthy population. Of the amount of this we can form no estimate; nor is it at all necessary to add more to prove that the sanatory improvements we advocate are alike demanded by real economy and true philanthropy.

To the economists another consideration may be urged. After orphanage and widowhood, sickness and weak health are the great causes of pauperism, and all these are increased by insalubrity. Anything which shall considerably diminish insalubrity, will therefore cause a very great diminution of pauperism, and consequent saving in poor-rate.

There may be some whom it is not undesirable to remind, that it is often far *cheaper* to prevent the causes of unhealthfulness, than to relieve the distress that it occasions. That it is far wiser to do so, all will agree.

